# Chelsea Hospital for Women

**Published:** 1902-06-07

**Authors:** 


### CHELSEA HOSPITAL FOR WOMEN.

### ANNUAL GENERAL MEETING.

THE attendance at this meeting, which took place on May 28th, was highly satisfactory.

The chair was taken by the Right Hon. Lord Glenesk, who congratulated the governors upon the success of the representations made to Lord Windsor's committee concerning the Tube Railway, which had resulted in the abandonment of the scheme for a tube railway to run under the Fulham Road. In conjunction with the other hospitals in the Fulham Road they had opposed the Bill, as the vibration caused by the tube would have had a very serious effect upon their hospital. Their operating room was at the top of the house, where the vibration would be most felt, and the slightest tremulousness during an important operation would be highly dangerous. Generally speaking, the comfort of the patients would have been considerably interfered with by the constant passing of trains. Lord Windsor's committee had taken all these points fully into consideration, and it was only fair to add that the promoters of the Bill had not urged its claim with very great persistence.

This matter being so happily settled they were now, continued Lord Glenesk, free to continue the work of the hospital without further anxiety, and the report would show how successfully the work had been carried on during the past year. Her Majesty the Queen and H.R.H. the Princess of Wales had consented to remain as Patron and Vice-Patron of the hospital. An important improvement had been carried out during the year 1901 in the shape of the reflooring of the wards, which had been undertaken on the recommendation of King Edward's Hospital Fund. Other improvements had also been carried out, so that the hospital must now be in a state very nearly bordering on perfection.

As regards the general appeal that was made all over London for King Edward's Hospital Fund, Lord Glenesk said that it should receive every encouragement, for by its means the multitude who were unable to pay the fees the medical men so thoroughly merited were enabled to be treated in a wise and scientific manner at the various hospitals. Each district ought to support its own hospital. They were assisting in this hospital by showing how good a hospital could be.

### THE YEAR'S WORK.

They had been well supported by their friends, and there had been a progressive number of severe cases treated, patients coming from all parts of the country, and even from the Colonies and the United States in consequence of the fame of the marvellous operations performed at their hospital. The deaths were under 2 per cent., which was quite an exceptional proportion, and was accounted for by the admirable arrangements of the hospital and the skill of the doctors. In 1901 the total of patients was 730, as against 673 in the previous year. During 1902 300 had already been admitted, which showed that the work was still going on. There was no need to appeal for money, the figures appealed for themselves. It was very delightful not to have to plead beggary or threaten to shut up the hospital as some were compelled to do. They needed money in order to undertake fresh work, but their finances were at the present time in a most satisfactory and thoroughly solvent condition. In consequence of the heavy expenses entailed by the re-flooring of the wards, there was a very slender margin available for the payment of accounts at the beginning of the year. This unsatisfactory state of things was, however, remedied through the generosity of their friend, H. M. E., who forwarded £500, which was reserved as an emergency fund. The same munificent donor later presented a further sum of £500, which was treated in the same way. They were particularly anxious not to have to touch this fund, but hoped to be able to add to it and thus make it into an endowment fund for the hospital.

The hospital had lost through death the help of Mr. T. W. Brookes, a trustee and member of the governing body, whose place was now filled by his brother.

With regard to the Convalescent Home at St. Leonard's, there was little to say except that it was doing a very good work, and going on in a very prosperous manner under its admirable matron.

The usual resolutions and votes of thanks were then adopted, Lord Glenesk proposing a special vote of thanks to H. M. E., one of their vice-presidents, for having so generously instituted an emergency fund. The meeting then separated.

